# Supplementary material for: Complexome profiling of the Chlamydomonas psb28 mutant reveals TEF5 as an early PSII assembly factor
Source: Plant Cell. 2025 Mar 17;37(6):koaf055. doi: 10.1093/plcell/koaf055 (PMC12164590; doi:10.1093/plcell/koaf055)
Supplement: koaf055_Supplementary_Data [file koaf055_supplementary_data.zip › psb28_interactive.zip!/plots/intensity/qualityPlots/peptides/_Cre01.g016514.t1.1.html]

### Cre01.g016514.t1.1

Trivial name:   
SeqCoverage [median]: 14.90  
Mass: 60.80 kDa  
  
 

| Sequence | MissedCleavage | Mass [Da] | wtIntensity | muIntensity | Score |
| --- | --- | --- | --- | --- | --- |
| AEELAKEQGFK | 1 | 1248.63 | 917.72 | 1515.41 | 72.65 |
| ALAEKEGDGMAK | 1 | 1218.59 | 4316.68 | 1352.32 | 84.29 |
| ALLAASGR | 0 | 757.44 | 2156.55 | 0.00 | 116.54 |
| DLASNIQGNLK | 0 | 1171.62 | 0.00 | 520.78 | 88.95 |
| DLASNIQGNLKR | 1 | 1327.72 | 399.26 | 6326.27 | 117.25 |
| MQVLDTAGK | 0 | 961.49 | 2317.18 | 1916.45 | 146.27 |
| VDIGGEVTAR | 0 | 1015.53 | 931.96 | 1544.01 | 101.65 |
| VTPGVPGVKPVVIELTDFK | 1 | 1994.15 | 529.99 | 0.00 | 72.68 |
| YGLPGRVDIGGEVTAR | 1 | 1658.87 | 0.00 | 1344.92 | 35.76 |
|  |  |  | 11569.3 | 14520.2 |  |
